# Larotrectinib treatment for infantile fibrosarcoma in newborns: a case report and literature review

**DOI:** 10.3389/fonc.2023.1206833

**Published:** 2023-07-27

**Authors:** Dandan Wang, Fanhui Zhang, Wanli Feng, Jiarong Pan, Tianming Yuan

**Affiliations:** ^1^ Department of Neonatology, Children’s Hospital, Zhejiang University School of Medicine, National Clinical Research Center for Child Health, Hangzhou, China; ^2^ Laboratory for Diagnosis and Therapy of Neonatal Diseases of Zhejiang Province, Hangzhou, China

**Keywords:** infantile fibrosarcoma, newborn, ETV6-NTRK3, larotrectinib, treatment

## Abstract

Infantile fibrosarcoma (IFS) is a rare tumor in childhood characterized by a single, localized, painless mass that grows rapidly but has a relatively indolent biological behavior and a favorable prognosis. Eighty-five percent of infantile fibrosarcomas are associated with t (12;15) (p13;25) chromosomal translocation resulting in ETV6-NTRK3 gene fusion, which provides the target for targeted therapy. Here, we report a case of IFS in a newborn with a mass in the left lower extremity confirmed by imaging, histopathological examination, tissue FISH testing, and high-throughput sequencing to detect gene rearrangement. Based on gene fusion targeted drug testing results, the patient was treated with standard doses of larotrectinib, resulting in significant mass shrinkage with no adverse effects, demonstrating the treatment effect of targeted therapy. This case provides a reference for using larotrectinib in newborns with IFS.

## 1 Introduction

Infantile fibrosarcoma (IFS) is a malignant tumor originating from fibroblasts in fibrous tissue and is the most common soft tissue tumor in children under one year of age, with 40% of cases present at birth (1). No standard treatment for IFS; surgical resection combined with chemotherapy is recommended. Recent studies revealed that over 85% of children with IFS exhibit a t (12,15)(p13;25) chromosomal translocation (2). This translocation leads to the fusion of the ETV6-NTRK3 genes, synthesizing fusion proteins that drive tumor development. In recent years, treatment with larotrectinib has shown good responsiveness and safety in treating IFS (3–5), but its use in newborns is rare. This article reports a case of larotrectinib treatment for IFS in a newborn.

## 2 Case presentation

**Figure 1:**
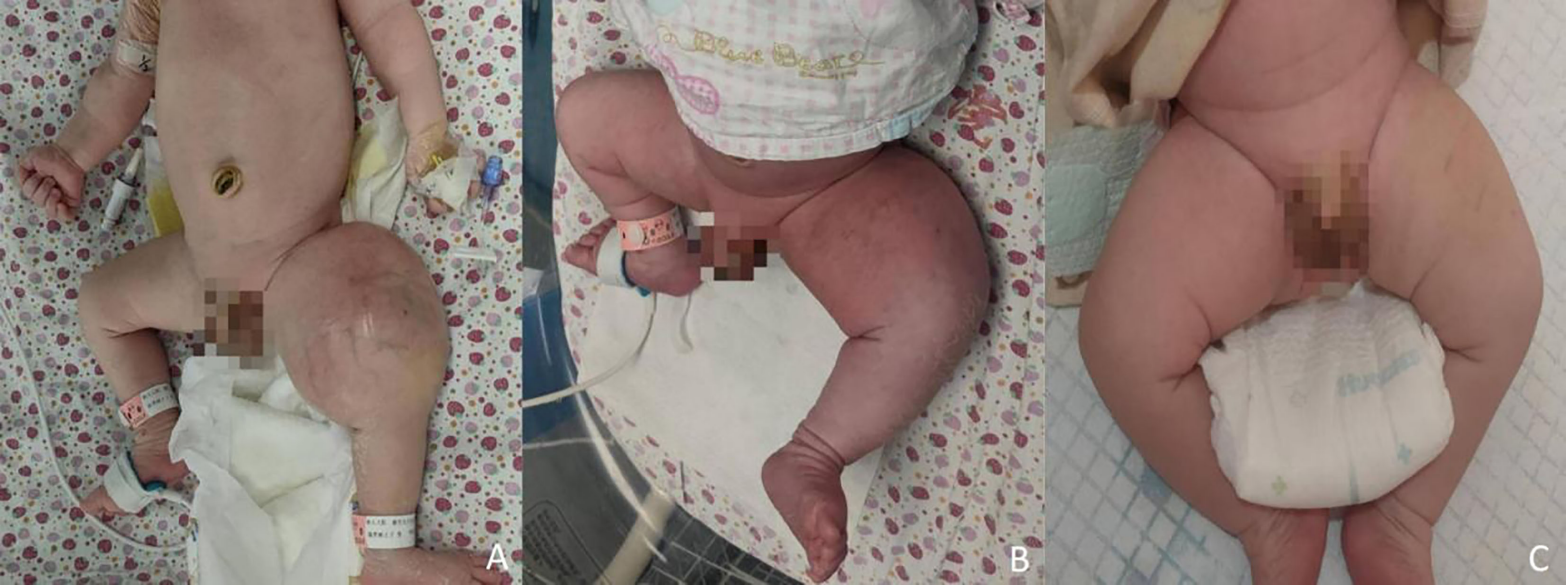
Changes in the left lower limb mass. **(A)** before treatment; **(B)** after ten days; **(C)** after one month.

**Figure 2:**
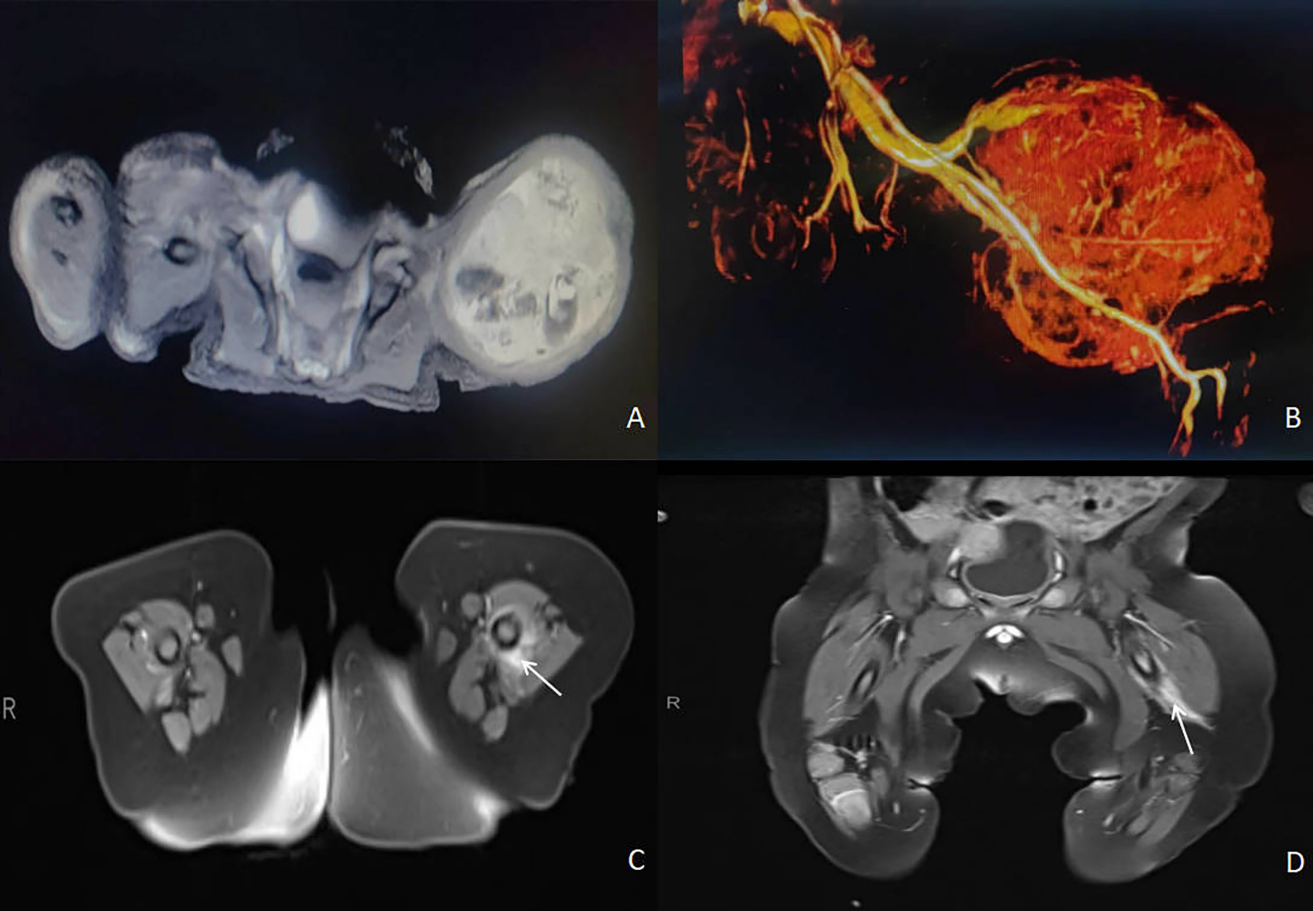
Imaging results. **(A)** MRI examination of the lower limb: a circular soft tissue mass in the left lower limb, measuring 8.25cm x 8.72cm x 7.86cm, closely adjacent to the femur and surrounding it, with unclear demarcation from the surrounding muscle tissue, and mixed-signal intensity in the mass. **(B)** enhanced CT of the lower limb: the mass is mainly supplied by the left femoral artery, with flux into the femoral vein, and many tortuous vessels are visible within the lesion. **(C, D)** MRI of the lower extremity revealed a significant mass reduction after four months of treatment.

**Figure 3:**
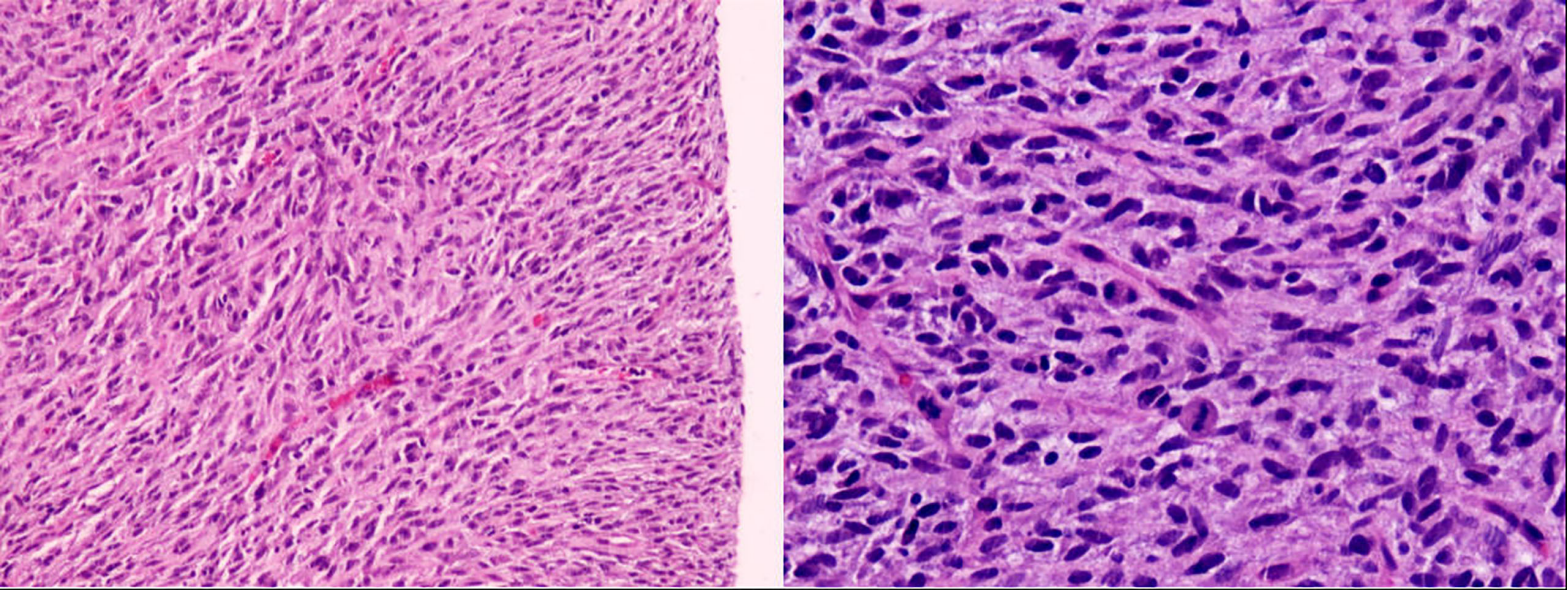
Pathology (microscopic examination shows spindle-shaped tumor cells with eosinophilic cytoplasm and frequent mitotic figures).

**Figure 4:**
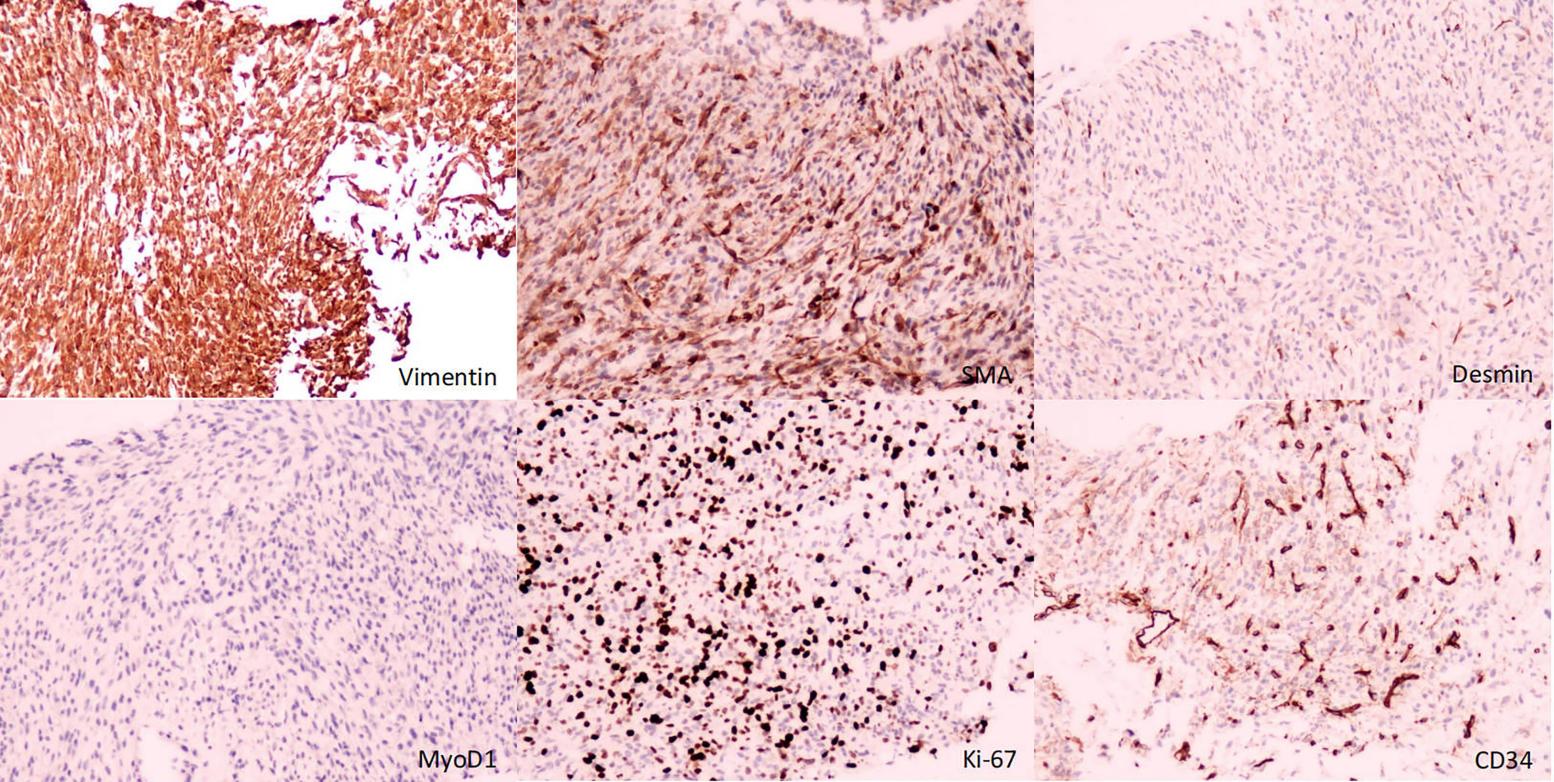
Immunohistochemistry (immunohistochemical monoclonal antibodies to Vimentin, SMA, Desmin, MyoD1, Ki-67, and CD34, respectively).

**Figure 5:**
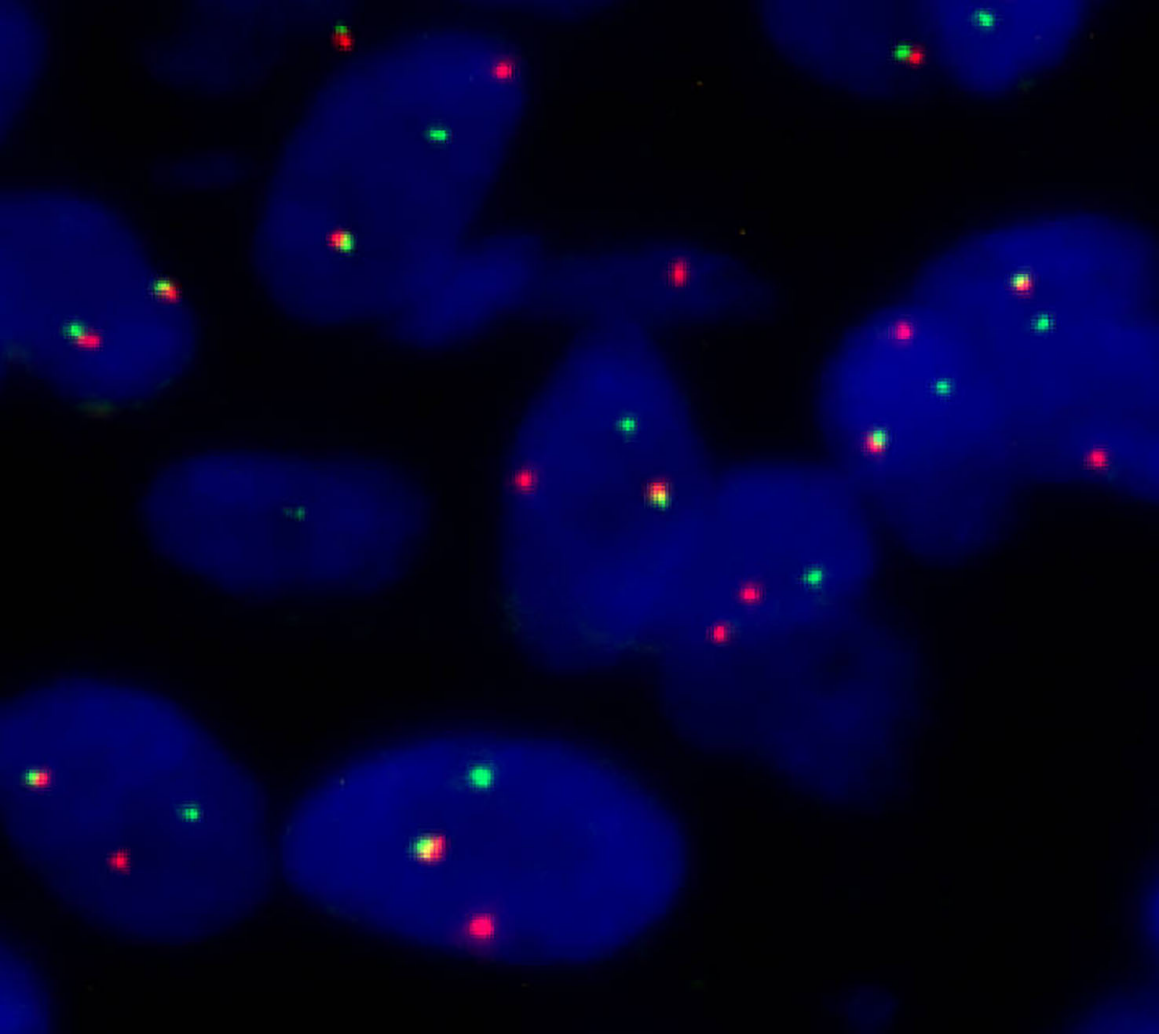
Fluorescence *in situ* hybridization (FISH) (separate red and green signals revealed ETV6 breakage).

A male newborn, born at a gestational age of 39 weeks and four days, was found to have swelling in the left thigh, with limited mobility at birth, and was admitted to Children’s Hospital, Zhejiang University School of Medicine. Physical examination revealed swelling in the left lower extremity, with a circumference of 29 cm (**Figure 1A**), local skin temperature, and limited mobility, while the right lower extremity had a circumference of 20 cm with normal mobility. After admission, magnetic resonance imaging (MRI) and enhanced CT scans of the lower limb were complete, and the results revealed a soft tissue mass in the thigh, measuring approximately 8.25 cm × 8.72 cm × 7.86 cm, with possible hemorrhage (**Figures 2A, B**. Subsequently, an ultrasound-guided fine-needle biopsy of the left thigh mass was performed under general anesthesia, and the histopathological results (**Figure 3**) suggested a soft tissue tumor with spindle cells. Immunohistochemical staining with monoclonal antibodies showed Desmin (weakly positive), CD34 (partially positive), EMA (focally positive), Pan-Trk (weakly positive), Vimentin (positive), INI1 (present), BRG1 (present), CD56 (partially positive), MyoD1 (negative), CD99 (positive), CK (AE1/AE3) (focally positive), Myogenin (negative), Ki-67 (approximately 30% positive), and SMA (positive) (**Figure 4**). Fluorescence *in situ* hybridization (FISH) revealed ETV6 gene breakage (**Figure 5**). High-throughput sequencing for gene rearrangement detection and targeted drug testing showed ETV6-NTRK3 rearrangement, and larotrectinib, entrectinib, and crizotinib were sensitive. The final diagnosis was infantile fibrosarcoma. At 20 days of age, oral larotrectinib (Vitrakvi, 50 ml/1.0 g) was started at a dose of 22 mg/dose twice a day (3.64 kg body weight, 100 mg/m^2^, twice a day). After ten days of treatment, the circumference of the left thigh decreased to 24.5 cm (**Figure 1B**). One month of follow-up showed a further reduction in circumference to 20 cm (**Figure 1C**), and ultrasound B revealed a mass size of 6.0 cm × 5.0 cm × 3.0 cm.

The patient has been followed for four months, and the tumor has shrunk significantly. But it still wrapped around the femur, and the boundary with the surrounding muscles is unclear(**Figures 2C, D**). The efficacy of larotinib therapy and the intricate nature of the tumor have prompted us to abandon any surgical approach at this time and maintain medical care until the tumor vanishes. We only considered surgery when larotinib resistance surfaced during treatment.

## 3 Discussion

IFS is a rare tumor that often presents as a single localized painless mass with rapid growth, most commonly found in the extremities and trunk and less commonly in the oral cavity, head and neck, colon, jejunum, periorbital, abdomen, and other sites (6–9). In this case, the mass of the newborn is located in the left lower limb, consistent with previous literature reports (10).

IFS must be differentiated from fibromatosis, hemangioendothelioma, rhabdomyosarcoma, lymphangioma, and neuroblastoma, but it isn’t easy to differentiate them based only on imaging examinations. We improved an aspiration biopsy of the lesion and histopathological examination to confirm the diagnosis, which showed spindle cells arranged in a fishbone-like pattern, with a high mitotic rate and focal necrosis visible under the microscope. Immunohistochemistry showed diffuse expression of Vimentin, minimal expression, or almost no expression of Desmin, CD34, and MyoD1. Tissue FISH examination confirmed the presence of ETV6 breakage, and high-throughput sequencing for gene rearrangement detection showed positive results for the ETV6-NTRK3 fusion gene. The detections mentioned above confirmed the diagnosis of IFS. However, it should be noted that the ETV6-NTRK3 fusion gene is not a completely specific marker for IFS, as this fusion gene has also been reported in patients with congenital mesoblastic nephroma, secretory carcinoma of salivary glands, secretory breast carcinoma, thyroid carcinoma, glioma, and some hematological disorders (11–16).

The current first-line infantile fibrosarcoma (IFS) treatment combines chemotherapy and surgery. However, chemotherapy can induce bone marrow suppression (17). Alkylating agents can cause renal and gonadal damage, while anthracycline drugs can lead to cardiac toxicity (10). Moreover, there are unique differences in the absorption, distribution, metabolism, and excretion of chemotherapy drugs in newborns, making the use of chemotherapy complex (18). Furthermore, IFS tumors are closely associated with adjacent blood vessels, nerves, or bones, posing a high risk of disability if complete surgical resection is attempted. Considering these factors, targeted therapy was used for a newborn with the ETV6-NTRK3 fusion gene. This fusion gene drives the expression of a fusion protein with sustained tyrosine kinase activity, acting as a driving factor in continuous downstream signal transduction, oncogenic transformation, and tumor growth (19). Larotrectinib, a tyrosine kinase inhibitor that works by blocking the altered fusion protein, is applicable for treating all types of solid tumors with NTRK gene fusion. The study demonstrated an overall response rate of 94% in paediatric patients with solid tumors containing fusions of the NTRK gene who received larotrectinib (20). Increasing evidence also supports the clinical benefits associated with larotrectinib (3, 4, 21, 22). However, caution should be exercised due to the potential presence of publication bias in the available reports. Treatment with standard doses of larotrectinib (100 mg/m^2^, twice a day) was started at 20 days of age, and after one month of treatment, the tumor size decreased from 8.25 cm × 8.72 cm × 7.86 cm to 6.0 cm × 5.0 cm × 3.0 cm. By the fourth month of treatment, the tumor had significantly decreased in size; however, an accurate measurement could not be obtained due to unclear demarcation between the tumor and surrounding muscle tissue. In summary, larotrectinib demonstrated favorable therapeutic efficacy in this patient, providing further evidence for its application in neonatal infantile fibrosarcoma.

Larotrectinib has been demonstrated to tolerate in pediatric patients, with most experiencing grade 1 or 2 adverse events. The most commonly observed adverse reactions include elevated alanine aminotransferase levels, leukopenia, neutropenia, vomiting, and other adverse events such as anemia, constipation, hypoalbuminemia, cough, and weight gain (5, 23). In this particular case, the patient has been followed up to date, and no adverse reactions have been observed. However, due to the relatively short duration of its current use, the long-term effects of larotrectinib on the growth and development of children have not been definitively determined.

A literature search using the keywords “infantile fibrosarcoma” and “larotrectinib” in the PubMed, Web of Science, and Embase databases revealed that this case is the second reported case of the use of larotrectinib for IFS in newborns since Caldwell KJ’s report (24). However, unlike Caldwell KJ’s case, larotrectinib was started at standard doses in this case rather than starting with a lower dose (1 mg/kg, twice a day) before escalating to standard doses. In our case, the visible deformity was no longer observed after one month, whereas Caldwell KJ’s reported case still had small deformities visible after six cycles. It is unknown whether the potential difference in dosage of Laroctinib is related to factors other than the size of the tumor itself. Our report suggests that standard doses of larotrectinib are safe for newborns with IFS.

Several literature reports on the issue of resistance to larotrectinib (5, 25, 26). The follow-up period for this case is relatively short, and the development of drug resistance is currently unclear. A longer follow-up period is necessary to gain more insight, highlighting a limitation of this particular case.

## 4 Conclusions

Pathological diagnosis is the gold standard for diagnosing IFS, and the ETV6-NTRK3 fusion gene helps confirm the diagnosis. Treatment with standard doses of larotrectinib is safe for newborns with IFS.

## Data availability statement

The original contributions presented in the study are included in the article/supplementary material. Further inquiries can be directed to the corresponding author.

## Ethics statement

Written informed consent was obtained from the individual(s), and minor(s)’ legal guardian/next of kin, for the publication of any potentially identifiable images or data included in this article.

## Author contributions

DW, FZ and WF reviewed the literature and contributed to manuscript drafting. TY and JP were responsible for revising the manuscript for important intellectual content. All authors contributed to the article and approved the submitted version.
